# A Mixed Method Survey of Characteristics of HIV Care Facilities: Medical Monitoring Project Facility Survey Project

**DOI:** 10.2196/52123

**Published:** 2025-05-29

**Authors:** Dustin Williams, John Weiser, Timothy McManus, Hanna B Demeke, Darryl Creel, Jason Craw, Milton Cahoon, Linda Beer

**Affiliations:** 1 RTI International Durham, NC United States; 2 Centers for Disease Control and Prevention Atlanta, GA United States

**Keywords:** multimode surveys, establishment surveys, imputation, weighting, nonresponse medical monitoring, HIV care, people with HIV, data collection, United States, Medical Monitoring Project, physicians, clinician, web surveys method, methods, HIV medical facilities, Americans, support, HIV epidemic, centers, mobile phone

## Abstract

**Background:**

Measuring the capacity of HIV medical facilities to deliver quality treatment and prevention care to people with HIV is essential to the over 1 million Americans living with HIV and supports federal efforts to end the HIV epidemic. To fill this gap and complement the ongoing Medical Monitoring Project—which conducts annual surveys of people with HIV and periodic surveys of HIV care providers—the US Centers for Disease Control and Prevention (CDC) and RTI (Research Triangle Institute) International conducted the Medical Monitoring Project Facility Survey (MMPFS).

**Objective:**

We aimed to describe the survey methods designed to achieve a high response rate from the 1022 facilities providing care to people with HIV as part of the Medical Monitoring Project—including frame development, survey instrument development, facility recruitment, and postsurvey data processing.

**Methods:**

For the MMPFS, the CDC and RTI developed a sequential multimode data collection approach (paper, web, and phone), including an abbreviated nonresponse follow-up (NRFU) instrument and the collection of administrative data for all facilities. Data were then processed to produce raw, imputed, and weighted datasets. Analyses included comparisons of responses to the full survey and NRFU survey.

**Results:**

The full MMPFS survey yielded 455 complete survey respondents and the NRFU survey yielded 59 complete survey responses, a combined response rate of 50.3% (514/1022). A nonresponse bias analysis comparing the 2 surveys found a significant difference in the raw datasets for 4 (12%) of the 34 categorical variables that were identical between the 2 surveys (all *P*>.0014). Weighted and imputed datasets were then generated and compared. There was no significant difference between the 2 datasets for any variable (all P>.05).

**Conclusions:**

The CDC and RTI’s MMPFS methodology proved to be a valuable means of collecting data from HIV care providers and providing estimates for facility characteristics related to the provision of health care for people with HIV. The combined response rate allowed the CDC and RTI to generate facility-level estimates and an imputed dataset that can be linked to MMPFS patient data. The methods may be applied to other facility survey studies.

## Introduction

Over 1.1 million Americans currently live with HIV, and many more are at risk of infection [1]. In 2019, the federal government launched the Ending the HIV Epidemic (EHE): A Plan for America initiative to end the US HIV epidemic by 2030. EHE focuses on early diagnosis, rapid and effective treatment for sustained viral suppression, preventing new transmissions using pre-exposure prophylaxis and syringe services programs, and responding quickly to outbreaks with prevention and treatment services for people who need them [2].

To guide the EHE, the National HIV/AIDS Strategy, and other national HIV prevention and care efforts, the US Centers for Disease Control and Prevention (CDC) conducts the Medical Monitoring Project (MMP). The MMP is a nationwide representative behavioral and clinical surveillance system of adults diagnosed with HIV in the United States, sponsored by the CDC [3]. The MMP is an annual, cross-sectional, complex sample survey with a 2-stage sampling design. In the first stage, 16 states (including 6 separately funded cities) and Puerto Rico were sampled, that is, from all US states, the District of Columbia, and Puerto Rico. In the second stage, simple random samples of persons aged ≥18 years with diagnosed HIV were drawn for each jurisdiction from the National HIV Surveillance System. Data are collected through phone or in-person interviews and medical records are abstracted at the most frequent source of HIV care during the past 24 months.

To complement the MMP and provide information to inform EHE activities, the CDC and RTI (Research Triangle Institute) International conducted the Medical Monitoring Project Facility Survey (MMPFS) to collect data on care delivery, patient engagement and retention practices, and workforce characteristics from facilities where MMP participants received HIV care. Specifically, the MMPFS surveyed all HIV care facilities at which a medical record abstraction took place during the MMP’s 2019 data collection cycle. The MMPFS collected data on the capacity of care facilities to deliver prevention care and services; provide HIV prevention messaging; partner with public health programs; offer services for HIV negative partners of people with HIV; engage and retain patients; and offer HIV pre-exposure prophylaxis, medication-assisted therapy, and other ancillary care and support services to those who need them. Information on facility location and workforce capacity was also collected to identify areas in need of expanded support to deliver these services. MMPFS data were also linked to MMP person-level data to enhance the understanding of facility-level facilitators and barriers and health care access and outcomes among people with HIV. Numerous studies have shown that HIV care facility characteristics—such as those related to funding, service provision, and workforce—play a role in access to services and shaping patient outcomes [4,5].

In this paper, we describe the MMPFS methods designed to achieve a high response rate and minimal nonresponse bias—including the creation of the frame of facilities surveyed; survey instrument development; data collection methods including the development of multiple survey modes and phases of data collection and facility recruitment; response rates; and postsurvey data processing, including nonresponse bias analysis, weighting, and imputation.

## Methods

### Instrumentation

The CDC and RTI attempted to collect data from 1022 HIV care facilities that provided HIV care during the MMP 2019 data collection cycle (June 2019 to May 2020) to a probability sample of adults with diagnosed HIV located in 23 MMP project areas: California (including the separately funded jurisdictions of Los Angeles County and San Francisco); Delaware; Florida; Georgia; Illinois (including Chicago); Indiana; Michigan; Mississippi; New Jersey; New York (including New York City); North Carolina; Oregon; Pennsylvania (including Philadelphia); Puerto Rico; Texas (including Houston); Virginia; and Washington. MMP participants identified these facilities as their most frequent source of HIV care during the past 24 months and were the place at which MMP medical record abstraction took place [3,6]. Human participants’ approvals were not obtained for the MMFS because respondents were staff of the facilities and no information on individuals was collected.

For the MMPFS, the CDC and RTI’s primary consideration was building survey instruments that offered busy physicians and facility administrators (targeted respondents for the MMPFS) a variety of options to reduce their response burden while maximizing response. To achieve this goal, the MMPFS full survey was programmed in web, mail, and phone modes, and a nonresponse follow-up survey (NRFU) was programmed in a web mode. The web surveys were programmed using Voxco (Voxco Group Inc) online to be easily viewed on a desktop or laptop computer, tablet, and smartphone to provide flexibility and reduce the burden for care providers and facility administrators with limited time to complete surveys. The phone survey was then programmed in Voxco’s computer-assisted telephone interviewing mode using the web survey as its base to minimize programming costs. The mail survey was programmed using TeleForm (Open Text Corporation) to minimize the time needed to capture mail survey data while improving accuracy.

Any of the 1022 sampled HIV care facilities from the 23 MMPS project areas that did not respond to the full survey were asked to complete the abbreviated MMPFS NRFU survey, which consisted of a subset of critical survey items and was estimated to take 5 minutes to complete. Additionally, we collected publicly available administrative data for all MMPFS facilities.

The full survey instrument was designed to document the provision of services that support the 4 pillars of the EHE: prevent, diagnose, treat, and respond. Survey domains included general characteristics (eg, facility type, sources of revenue, patient volume, and provider staffing levels), HIV testing and pre- and postexposure prophylaxis provision, clinical and supportive services provided onsite and through established referral relationships, capacity for supporting rapid enrollment and initiation of antiretroviral therapy and retention in care, collaboration with health departments on public health activities, availability of telehealth visits, and policies to prevent patient exposure to COVID-19. The survey instrument was developed in collaboration with MMP provider and community advisory board members, selected HIV care facility leaders, and the CDC and other federal agency subject matter experts.

### Ethical Considerations

Because respondents were staff of the facilities and no information was collected on individuals, human participants’ approval was exempted by the RTI’s Institutional Review Board (ethics exemption approval: STUDY00021321). All responding facility data were maintained in a secure environment that could only be accessed by project staff. All publications of data were deidentified. Responding facilities were offered no compensation for their participation.

### Frame Development

Concurrent with MMPFS instrumentation, the CDC and RTI worked with MMP project areas to develop and refine the MMPFS frame. MMPFS frame development began in October 2020 with the development of a MMPFS mailing list upload site for MMP project areas to upload a workbook containing data for all facilities providing care to MMP participants during the 2019 MMP data collection cycle. From October 2020 through April 2021, MMP project areas uploaded workbooks containing facility information (name, geographic location, and mailing address), primary contact information (name, title, phone number, and email address), and secondary contact information (name, title, phone number, and email address) for each facility.

**Figure 1:**
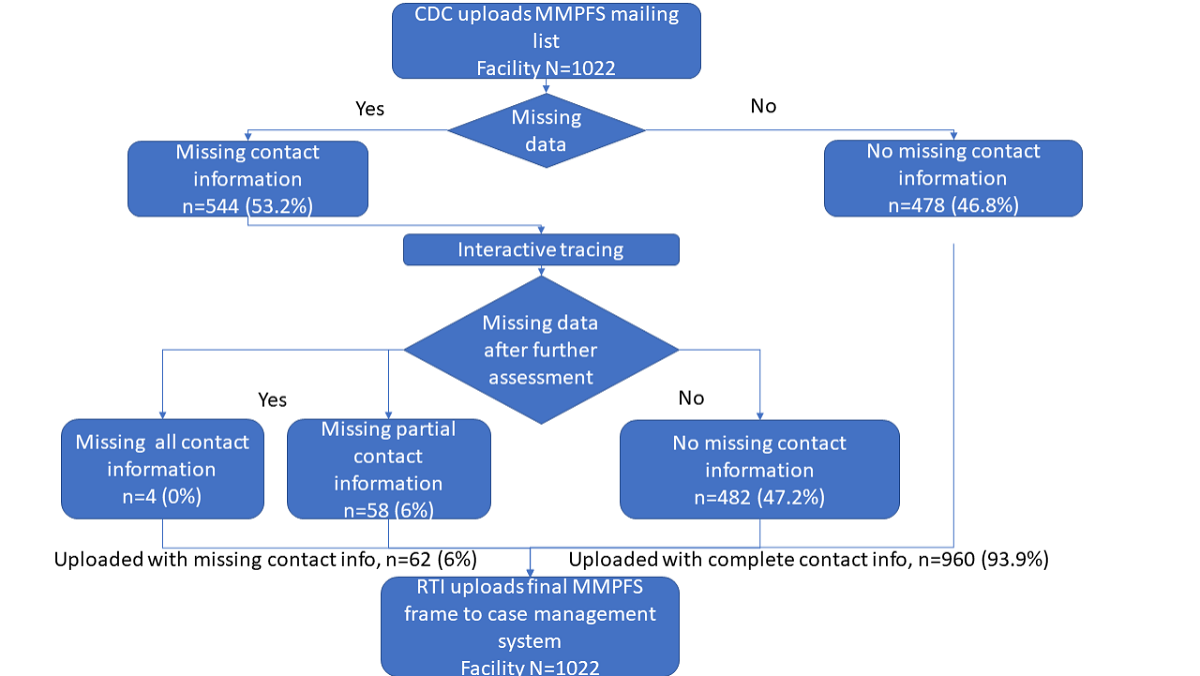
Medical Monitoring Project Facility Survey interactive tracing results (N=1022). CDC: Centers for Disease Control and Prevention; MMPFS: Medical Monitoring Project Facility Survey; RTI: Research Triangle Institute.

Due to high levels of item missingness for primary contact name and email address information, the CDC and RTI conducted web-based tracing on 544 facilities with missing frame data from May to June 2021. During web-based tracing, the RTI telephone interviewers conducted web searches to identify all missing information for each facility. After locating missing information, telephone interviewers then conducted for up to 5 call attempts to verify the accuracy of information identified via web search. Using web-based tracing, the CDC and RTI were able to locate sufficient contact information for mail and phone recruitment for nearly all MMPFS facilities. After web-based tracing, of the 1022 MMPFS facilities, 960 had no missing contact information, 58 had a partial facility record (missing some, but not all contact information), and 4 were missing all contact information. All 1022 cases were loaded into the MMPFS case management system with a unique case ID after web-based tracing—results from web-based tracing are presented in Figure 1.

### Survey Recruitment

For the MMPFS full survey, the CDC and RTI used a modified version of Don Dillman’s Tailored Design Method to recruit facilities to participate using a variety of recruitment methods and varied mailing types [7].

MMPFS recruitment began with mail and email invitations followed by a reminder postcard and 3-week mail and email reminders—the 4 facilities missing all contact information were excluded from mail and email recruitment activities but were included in response rate calculations. Given the slower-than-expected response after the 3-week reminders, the CDC and RTI worked with the Health Resources and Services Administration, HIV Medicine Association, and Association of Nurses in AIDS Care to develop an email and letter endorsing the MMPFS that leaders of these 3 agencies cosigned. The 3-agency letter of support email was sent 2 weeks after the 3-week reminders. In the 2 weeks following the letter of support email, the number of completed full surveys increased from 176 to 278, which suggests this email had a positive impact on recruitment.

The 3-agency letter of support was included in the 7-week mail and email reminders. After all mail and email outreach was completed for the full survey, phone outreach was initiated to nonresponding agencies.

MMPFS phone recruitment began on September 14, 2021, lasting through October 22, 2021. During phone survey prompting, a facility received up to 5 call attempts—unless they completed the survey during a prior call attempt, completed a survey via full web or mail survey between call attempts, or refused to participate during a call attempt. Overall, a total of 707 facilities received call attempts. While only 1 phone survey was completed during phone prompting, an additional 219 cases were completed via the web or mail modes following phone prompting, which suggests this was an effective recruitment tactic for the web and mail modes.

After completion of the full survey recruitment, the CDC and RTI launched the MMPFS NRFU survey. NRFU surveys are surveys where the nonrespondents (refusals or noncontacts) are asked to complete a shorter questionnaire [8]. The MMPFS NRFU survey was critical for ensuring the MMPFS represented the full population of HIV facilities at which MMP participants received care. For example, if certain types of MMP facilities responded at lower rates than other facilities, it could introduce bias. The MMPFS NRFU survey was designed to determine whether nonresponding facilities differed systematically from responding facilities on key survey data elements and mitigate any potential biases.

From October 18, 2021, to October 25, 2021, depending on the date that phone recruitment ended for the facility, the CDC and RTI sent a NRFU web survey invitation email followed by a reminder email to all 490 facilities that had not responded or refused to participate.

In addition, on October 21, 2021, a list of all nonresponding agencies in each project area’s jurisdiction was made available for each project area and the CDC asked project areas to encourage nonresponding facilities to complete the MMPFS NRFU survey. Before this outreach, there were 674 pending facilities and there were an additional 33 completed full surveys at the end of the full survey field period without additional outreach. The CDC and RTI found the number of additional completed full surveys to be greater than anticipated at this point in data collection (and with NRFU data collection ongoing) and believe the advocacy of MMP project areas helped boost response, with 111 respondents completing after the start of this recruitment activity. Additional experimentation would be needed to determine the efficacy of such interventions for other surveys, but this suggests that engaging entities that have established relationships with facilities in recruitment activities (as was the case for the MMP project areas) increases response rates.

Additional details on MMPFS full and NRFU survey recruitment activities and completion rates before each activity are shown in Tables 1 and 2—completion rates use a denominator of 1022 and include the 4 facilities that did not receive recruitment correspondence due to missing contact information.

**Table 1:** MMPFS^a^ recruitment milestones by date, type, facilities receiving outreach, and cumulative response and completion percentage on the date of recruitment activity: full survey.

Event	Date	Facilities receiving recruitment outreach	Cumulative responses on the date of recruitment activity (N=1022), n (%)	Notes
MMPFS email invitation	July 23, 2021	1018	0 (0)	—^b^
MMPFS mail invitation	July 23, 2021	1018	0 (0)	Invitation letter, paper survey, and business reply envelope
MMPFS reminder postcards	July 30, 2021	975	43 (4.2)	—
MMPFS 3-week reminder email	August 13, 2021	914	104 (10.2)	—
MMPFS 3-week reminder letter	August 13, 2021	914	104 (10.2)	Reminder letter, paper survey, and business reply envelope
HRSA^c^/HIVMA^d^/ANAC^e^ support email	August 25, 2021	842	176 (17.2)	—
MMPFS 7-week reminder email	September 10, 2021	740	278 (27.2)	—
MMPFS 7-week reminder letter	September 10, 2021	740	278 (27.2)	Reminder letter, HRSA/HIVMA/ANAC letter of support, paper survey, and business reply envelope
MMPFS phone nonresponse prompting	September 13, 2021, to October 25, 2021	707	311 (30.4)	Up to 5 call attempts
MMPFS project area outreach	October 21, 2021, to November 15, 2021	674	344 (33.7)	—
MMPFS data collection closes	November 15, 2021	—	455 (44.5)	—

^a^MMPFS: Medical Monitoring Project Facility Survey.

^b^Not applicable.

^c^HRSA: Health Resources and Services Administration.

^d^HIVMA: HIV Medicine Association.

^e^ANAC: Association of Nurses in AIDS Care.

**Table 2:** MMPFS^a^ recruitment milestones by date, type, facilities receiving outreach, and cumulative response and completion percentage on the date of recruitment activity: NRFU^b^ survey.

Event	Date	Facilities receiving recruitment outreach	Cumulative responses (N=1022), n (%)
MMPFS NRFU survey email invitations	October 18, 2021, to October 25, 2021	490	0 (0)
MMPFS NRFU survey reminder email	November 8, 2021	452	38 (3.7)
MMPFS data collection closes	November 15, 2021	0	59 (5.8)

^a^MMPFS: Medical Monitoring Project Facility Survey.

^b^NRFU: nonresponse follow-up.

### Data Processing

In November 2021, the CDC and RTI conducted web searches to collect publicly available administrative data for all 1022 facilities, including Federal Information Processing System code, primary care health professional shortage area designation, medically underserved area or population designation, Rural-Urban Continuum Code, and Ryan White HIV/AIDS Program funding type. In addition, the CDC provided the number of abstractions performed at each facility during the 2019 MMP data collection cycle to be included in the minimal dataset. The goal of the minimal dataset was to fill the remaining gaps in the survey data on critical items (eg, Ryan White Program funding), improve survey estimates, and provide valuable information for nonresponse adjustments.

After completing the minimal dataset, a dataset for the full MMPFS survey was produced. The full survey dataset contained data on 155 analytic variables for all 1022 responding facilities—only frame and minimal dataset data were included for full survey nonresponders.

A raw dataset was then produced for the MMPFS NRFU survey. The NRFU dataset only included the 567 facilities that did not complete a MMPFS full survey. The MMPFS NRFU dataset did not include full survey respondents to allow for comparisons between the responders in the 2 datasets. The MMPFS NRFU dataset had 36 analytic variables—only frame and minimal dataset data were included for NRFU survey nonresponders.

After producing the raw datasets, data quality checks and editing procedures were applied to both datasets, including checks for valid values, correct codes assigned for missing and unknown data, checks for skip pattern consistency and appropriate coding of skipped variables, and appropriate coding of missing variables from broken off or partial surveys.

All cases were then assigned a final status code based on response type (full complete, full partial, NRFU complete, NRFU partial, noncontact, and noncooperation) and the number of valid responses for a facility—the number of valid responses excludes the address verification question as a possible response variable. Facilities in the eligible, noninterview noncontact status group did not attempt to complete a web, mail, or phone survey for the full or NRFU surveys. Facilities in the eligible, noninterview noncooperation status groups accessed a full or NRFU survey but did not provide any valid responses beyond the address verification question. The Full Survey Respondent status group had at least one valid response and a full survey complete response—excluding address confirmation. The NRFU Survey Respondent status group had at least one valid response and a NRFU survey complete response. The MMPFS full and NRFU surveys did not have any ineligible facilities. That is, all facilities were deemed eligible at the start of this study because they provided care to a 2019 MMP participant.

### Nonresponse Bias Analysis

The CDC and RTI then conducted a nonresponse bias analysis on the MMPFS data. The goal of the nonresponse bias analysis was to identify the best variables to use for postdata collection weight adjustments, which use variables that are associated with response propensity and key outcome variables [9]. For the MMPFS, the RTI and CDC wanted to assess whether certain HIV care facilities’ characteristics (funding, types of health coverage or insurance accepted, HIV caseload, and staff training); onsite clinical and support services or referral agreements for off-site services; rapid linkage to HIV care and antiretroviral therapy initiation practices and barriers; use of HIV telemedicine; use of data to systematically monitor retention of care; and technology to increase retention of care were associated with response propensity.

First, full survey and NRFU survey data were compared for the frame and minimal dataset variables to compare differences in facility types or operations. Second, the CDC and RTI compared the responses for the full survey respondents to the NRFU survey respondents for the 35 analytic variables on both datasets to compare responses. Further, 34 of the variables were categorical and 1 variable was continuous. Categorical variables were analyzed by comparing the proportions with a value of 1, usually the “yes” category. Instead of viewing the 35 tests as individual tests at α equal to .05, we viewed the 35 tests as a single “family” of tests and controlled the familywise error rate to minimize the probability of making false discoveries. To account for the multiple testing conducted, the α levels were adjusted for significance using the Bonferroni adjustment, which is α divided by the number of tests. For MMPFS, it was assumed the α was equal to .05 and the Bonferroni adjusted α is .05/35 (approximately .0014). Therefore, for the MMPFS, observed *P* values had to be less than .0014 to be statistically significant. Bonferroni is a conservative approach to the multiple comparison problem, but, if we were to err, we wanted to err on the side of not making a false discovery (ie, decrease the chance of a type I error, with a possible increased chance of a type II error). In addition, the RTI ran a Holm-Bonferroni adjustment, which yielded the same results.

### Postsurvey Weight Adjustments

For the MMPFS postsurvey weight adjustments, we first compared the number of abstractions completed at each facility during the 2019 data collection cycle with the patient with HIV load size reported on the full survey question. Based on this comparison, no clear association was identified between the reported patient load and number of MMP abstractions completed, indicating that the number of MMP abstractions completed was not an accurate measure of facility size.

Next, postsurvey weight adjustments were calculated so that the 455 full survey respondents would represent the 1022 facilities. The postsurvey weight evaluation started with all 9 frame and minimal dataset variables in the weight adjustment model (not counting the number of abstractions that had been previously eliminated). Variables were then removed using backward elimination, with the variable with the largest *P* value greater than .05 being removed from the model. The backward elimination modelling process was repeated until all variables in the weight adjustment model had *P* values less than or equal to .05. Table 3 shows the variable description and type for 3 Ryan White HIV/AIDS Program funding variables, frame data on MMP abstractions (other frame data was respondent contact information, which was not of interest), and all minimal dataset variables included in the weight adjustment model—this includes all minimal dataset variables. Finally, the CDC and RTI used the cross-classification of the 3 Ryan White variables to create the weight adjustments and final weights.

**Table 3:** Medical Monitoring Project Facility Survey weight adjustment types by variable types and inclusion in the weight adjustment model

Description	Variable type	Included in weight adjustment model
Number of Medical Monitoring Project abstractions at this facility (a proxy for the size of the facility)	Continuous	No
Primary health care professional shortage area	Binary	No
Medically underserved area or population	Binary	No
Rural-urban continuum code	Ordinal (categories 4-9 into a single nonmetro county category)	No
Does the facility receive Ryan White HIV/AIDS program funding?	Binary	Yes
Does the facility receive Ryan White HIV/AIDS Part A program funding?	Binary	Yes
Does the facility receive Ryan White HIV/AIDS Part B program funding?	Binary	No
Does the facility receive Ryan White HIV/AIDS Part C program funding?	Binary	No
Does the facility receive Ryan White HIV/AIDS Part D program funding?	Binary	No
Does the facility receive Ryan White HIV/AIDS Part F program funding?	Binary	Yes

### Imputation

The CDC and RTI then performed imputation for nonresponding MMPFS facilities. Imputation allowed the CDC to link facility characteristics to MMP patient-level data for all MMP patients. The imputation methodology consisted of 2 steps: recursive partitioning (trees) to create imputation classes and then a weighted sequential hot deck to produce the imputed values. For each variable or vector of variables imputed, a prediction tree was constructed from the respondents. The terminal nodes of the tree are the imputation classes and every respondent was in an imputation class. Once the tree was constructed, we ran the nonrespondents through the tree so they could be placed in an imputation class. After the nonrespondents were placed in an imputation class there was a respondent and nonrespondent imputation class. Next, the weighted sequential hot deck was implemented within each imputation class. The final weight for the full survey respondents was used for their imputations, and a weight of 1 was used for all facility imputations. For imputation, Ryan White Funding and the node were used as class variables; and Ryan White Part A Funding, Ryan White Part F Funding, and the number of patients for whom the facility provided HIV care during the past year were used as sorting variables. If the cross-classification of Ryan White Funding and node created an imputation class without any donors, the minimum size of the nodes was increased until every node had donors.

The imputation methodology was applied in three steps:

1. Used data from donors (completed survey responses from facilities that had similar frame and minimal dataset characteristics) to impute missing items on the full survey respondent dataset for nonrespondents.
2. Imputed missing items on the NRFU respondent dataset. For items on the full and NRFU surveys, missing NRFU values were imputed using donors (respondents) from the full and NRFU survey data. For items not on the NRFU survey, answers were imputed using data from full survey respondents.
3. Imputed all items for all nonrespondents using the full survey and NRFU imputed dataset respondents.

The imputed and weighted survey estimates were then compared using the weighted survey estimate and imputed survey estimate to develop a contrast (difference) survey count, contrast survey estimates, contrast SE, contrast 95% CI lower limit, contrast 95% CI upper limit, and contrast *P* value.

## Results

### Instrumentation

For the MMPFS, 455 respondents completed full surveys (253 web surveys, 201 mail surveys, and 1 phone survey) and 59 completed a NRFU web survey of the entire sample of 1022 facilities. The final combined final response rate was 50.3% (514/1022)—the full survey response rate was 44.5% (455 full survey responses out of 1022 sampled facilities) and the NRFU survey response rate was 5.8% (59 NRFU survey responses out of 1022 sampled facilities) of the total sample. While NRFU response rates are expected to be low, the low NRFU response rate suggests that the length of the full survey may not have been the reason for not responding to the full survey for many facilities.

### Survey Recruitment

The full MMPFS survey was open from July 23, 2021, to November 15, 2021, and allowed facilities to complete the survey across 3 modes: web, mail, or phone. Overall, a total of 260 full web surveys were submitted, including duplicate and partial web surveys; 253 were conducted on desktop computers and 7 on smartphones. The average completion time for the full web survey was 14 (SD 11) minutes. The mail survey was conducted simultaneously and a total of 201 MMPFS mail surveys were completed. The phone survey was open from September 13, 2021, to November 15, 2021, and a total of 1 phone survey was completed.

The MMPFS NRFU survey was open from October 18, 2021, to November 15, 2021, and allowed facilities to complete an abbreviated web survey. Overall, a total of 59 surveys were submitted via the web NRFU survey. For the NRFU web survey, 54 were conducted on desktop computers and 5 on smartphones. The average completion time for NRFU web surveys was 6 (SD 4) minutes. Details on survey completion by mode are included in Tables 4 and 5. In addition, 111 responses were received after project area outreach started, which suggests that the personal and professional relationships the project areas have with facility administrators might have been a powerful tool for encouraging response.

Table 6 contains a summary of MMPFS facilities by status group.

**Table 4:** Medical Monitoring Project Facility Survey responses and completion rate by mode: full survey.

Event	Responses by mode (N=1022), n (%)
Web survey	260 (25.4)
Mail survey	194 (19)
Phone survey	1 (0.1)
Full survey total	455 (44.5)

**Table 5:** Medical Monitoring Project Facility Survey responses and completion rate by mode: NRFU^a^ survey.

Mode	Cumulative responses (N=1022), n (%)
Web survey	59 (5.8)
NRFU survey total	59 (5.8)

^a^NRFU: nonresponse follow-up.

**Table 6:** Medical Monitoring Project Facility Survey response count, percent, cumulative count, and cumulative percentage by final contact status groups (N=1022).

Status group	Value, n (%)	Cumulative value, n (%)
Eligible, noninterview noncontact (never accessed either survey)	474 (46.38)	474 (46.38)
Eligible, noninterview noncooperation (accessed a survey, but did not provide any valid responses)	34 (3.33)	508 (49.71)
Full survey respondent	455 (44.52)	963 (94.23)
Nonresponse follow-up survey respondent	59 (5.77)	1022 (100)

Next, MMPFS contact, cooperation, and response rates were calculated. For the MMPFS, contact rates were calculated by dividing the number of respondents who made any attempt to complete a full or NRFU survey (n=548), including break offs, by the total sample (N=1022). The cooperation rate is then calculated by dividing the number of completed surveys by the number of contacted respondents who provided 1 valid response beyond the address verification question for each survey, using the number of contacted respondents (n=548) for the denominator on both surveys. For the MMPFS surveys, the response rate is the product of the contact and cooperation rates. Table 7 shows 3 different rates (contact, cooperation, and response rates) for 3 different groups of respondents (combined full and NRFU survey respondents together, only full survey respondents, and only NRFU survey respondents).

**Table 7:** Medical Monitoring Project Facility Survey contact counts, contact rates, cooperation counts, cooperation rates, and response rates by respondent type (N=1022).

Group	Contact (N=1022), n (%)	Cooperation (n=548), n (%)	Response rate (contact × cooperation; %)
Full and NRFU^a^ survey respondents	548 (53.6)	514 (93.8)	51
Full survey respondents	548 (53.6)	455 (83)	45
NRFU survey respondents	548 (53.6)	59 (10.8)	6

^a^NRFU: nonresponse follow-up.

### Nonresponse Bias Analysis

There was no statistically significant difference for 30 (88%) of the 34 categorical variables tested (all *P*>.0014), and 2 of the differences were where the NRFU survey estimate was 0, meaning the MMPFS full survey data provides essentially the same results as analyzing the MMPFS full and NRFU survey combined data. Table 8 shows the variable description, full survey estimate, NRFU survey estimates, contrast (difference) estimate, contrast SE estimate, contrast 95% CI lower limit, contrast 95% CI upper limit, and contrast *P* value for the 4 statistically significant MMPFS analytic variables—those with observed contrast *P* values smaller than .0014 (.0005, .0009, .0005, and .0004), the Bonferroni adjusted α. Given that the vast majority of the variables were not statistically significantly different between the datasets—meaning analysis of the MMPFS full survey data alone provides essentially the same results as analyzing the MMPFS full and NRFU survey combined data—the CDC and RTI decided not to use the NRFU data for the weighted facility estimates.

**Table 8:** Medical Monitoring Project Facility Survey full survey and NRFU^a^ survey variable estimates, contrast survey counts, contrast estimates, contrast SE estimates, contrast 95% CI lower and upper limits, and contrast *P* values for variables with a significant difference (.0005, .0009, .0005, and .0004) between the full and NRFU survey estimates.

Variable description	Full survey estimate	NRFU survey estimate	Contrast survey count^b^	Contrast estimate	Contrast SE estimate	Contrast 95% CI limit	Contrast *P* value
Is the facility a federally qualified health center look-alike?	2.65	0	511	–2.65	0.76	–4.14, –1.17	.0005
Does the facility accept no common types of health coverage?	2.44	0	509	–2.44	0.73	–3.88, –1.01	.0009
Is there normally a physician at the facility at least 5 days per week who can provide HIV care?	77.03	91.53	503	14.5	4.14	6.36, 22.64	.0005
Does the facility provide onsite substance use disorders treatment?	31.54	55.93	506	24.39	6.83	10.96, 37.81	.0004

^a^NRFU: nonresponse follow-up.

^b^Number of combined responses from full and nonresponse follow-up surveys for each variable.

### Postsurvey Weight Adjustments

Table 9 shows the final weights for all 1022 facilities, including 567 facilities with a weight of 0 (all nonrespondents to the full survey, which includes MMPFS NRFU survey respondents). The remaining 455 facilities are the full survey respondents with weights ranging from 1 to approximately 3 with an unequal weighting of ~1.1153. This results in an estimate of the effective sample size—the sample size divided by the design effect (for this study, the unequal weighting effect)—of approximately 408 facilities (455 facilities/1.1153).

**Table 9:** Final survey weights for Medical Monitoring Project Facility Survey (N=1022).

Final weight	Value, n (%)	Cumulative value, n (%)
0.0000	567 (55.48)	567 (55.48)
1.0000	94 (9.2)	661 (64.68)
1.8229	96 (9.39)	757 (74.07)
2.5522	67 (6.56)	824 (80.63)
2.9394	198 (19.37)	1022 (100)

### Imputation

The comparison of the imputed and weighted data revealed that none of the contrast *P* values were statistically significant (all *P*>.05) Therefore, using either the imputed or weighted dataset would generate essentially the same point estimates.

The critical difference between the MMPFS imputed and weighted datasets is that the SEs for the imputed data are roughly 60% of the SEs of the weighted data, which could lower the *P* values for analyses performed using the imputed dataset. This is because the imputed dataset has about twice as many facilities as the weighted dataset, has no variability in the weights, and is treated as observed respondent data. That is, the imputed data do not account for the uncertainty in the imputed values. Due to the weighted data having generally larger SEs, a conservative approach to facility-level data analysis would be to use the weighted dataset. An alternative would be to conduct multiple imputation, which creates better estimates of SEs by accounting for differences in the SEs from the multiple imputations. However, multiple imputation was not used because it would require creating multiple datasets for the facility analysis and complicate patient-level analysis that would use the multiple imputed facility datasets. That is, each of the *M* multiple imputed facility datasets would have to be merged to the patient-level dataset to create *M* merged facility or patient datasets to conduct the analysis, which could introduce difficulties for users trying to conduct patient-level analysis using facility characteristics.

Despite the smaller SEs for facility-level estimates using the single imputation dataset, we concluded that imputed facility values could be validly appended onto the patient-level MMP dataset without impacting patient-level variance estimates because those are based on the patient-level dataset and its structure. That is, the imputed facility data used for patient-level analysis are used as characteristics of patients and not as facility-level analytic variables.

## Discussion

The CDC and RTI’s MMPFS methodology proved to be a valuable means of collecting data from HIV care facilities and providing estimates for critical factors related to the provision of health care for people with HIV. The methodology yielded a 50.3% response rate—which is relatively high for establishment surveys—with minimal nonresponse bias by using the described frame development, instrument development, data collection methods, facility recruitment, and postsurvey data processing methods. The combined response rate allowed the CDC and RTI to generate facility-level estimates and an imputed dataset that can be linked to MMPFS patient data.

These methods may be applied to other surveys of HIV care facilities, medical establishments, and health care providers, as this methodology used innovative methods (eg, the NRFU survey) and demonstrated ways to maximize response. This is significant given the lack of publications on establishment surveys, broadly, and medical establishment surveys, specifically. These types of surveys are critical for public health but often use methods designed and tested for household surveys.

The 50.3% combined response rate was notable given the potential negative impacts on facility response due to the ongoing COVID-19 pandemic during MMPFS data collection and might be partially attributed to the multimode design. The MMPSFS also allowed the CDC and RTI to generate facility-level estimates and an imputed dataset that can be linked to MMPFS patient data. Additionally, MMPFS results suggest that there may be a benefit to using letters of support from federal agencies and professional organizations and the endorsement of local health departments to boost participation in surveys of medical facilities, which is worth further study.

While the minimal dataset for the MMPFS only yielded 3 weight adjustment variables, these variables were valuable in producing datasets that could be linked to patient data. Future surveys of medical establishment and health care providers should consider the variables used by the MMPFS and explore additional sources of administrative data to identify other significant variables that could be used to improve future weighting and imputation efforts.

In addition, if there are future iterations of the MMPFS (or similar studies of health care providers and medical establishments), the costs and benefits of the NRFU survey should be reconsidered, given the costs of the NRFU survey and that there were few significant differences (*P*=.0005, .0009, .0005, and .0004) between the full survey and NRFU survey data for this iteration. If there are significant changes in the facility characteristics and provision of care for people with HIV (eg, more MMP facilities are part of large health networks at the time of survey administration), it may be worth conducting another NRFU survey. However, if there are few changes in the population and a similar response is elicited from the initial survey, it may be prudent to forego a NRFU survey and use those resources to boost response (eg, additional recruitment activities).

A limitation of this assessment is that we did not perform any formal experiments to test the MMPFS methodology. In the future, it would be useful to design formal experiments on medical establishment and health care provider surveys to better understand the effects of time between survey outreach events and response rates. For busy medical facilities that may take longer to process noncritical mail than households or other establishments, it could be useful to test whether more time between mailings and a longer data collection period yield a higher response rate. It may also be useful to test the effect of the use of “special mailing” types (eg, Federal Express or Priority Mail) on medical facility response. Such special mailings may be more likely to stand out to overburdened medical facilities and generate an improved response rate. In addition, we did not conduct a formal assessment of survey costs, which could be helpful for the implementation of MMPFS methods in other settings.

The MMPFS methodology proved valuable in collecting data on care delivery, patient engagement and retention practices, and workforce characteristics from facilities where MMP participants received HIV care. This data will provide information that will help inform future EHE activities. In addition, MMPFS methods can be used to inform and improve response on future surveys of HIV care facilities, medical establishments, and medical providers. Specifically, the findings illuminate the benefits of using letters of support, multimode and multiphase data collection approaches, and the use of administrative data for data processing and analysis activities.

## Abbreviations

CDC: Centers for Disease Control and Prevention
EHE: Ending the HIV Epidemic
MMP: Medical Monitoring Project
MMPFS: Medical Monitoring Project Facility Survey
NRFU: nonresponse follow-up
RTI: Research Triangle Institute

## Data Availability

Data are not publicly available due to security and confidentiality guidelines governing the collection of public health surveillance data. Restricted access may be granted on request from the CDC.
